# Gridded land use data for the conterminous United States 1940–2015

**DOI:** 10.1038/s41597-022-01591-0

**Published:** 2022-08-13

**Authors:** Caitlín Mc Shane, Johannes H. Uhl, Stefan Leyk

**Affiliations:** 1 Department of Geography, University of Colorado Boulder, 260 UCB, Boulder, CO 80309 USA; 2 Cooperative Institute for Research in Environmental Sciences, University of Colorado Boulder, Boulder, CO 80309 USA; 3 Institute of Behavioral Science, University of Colorado Boulder, Boulder, CO 80309 USA

**Keywords:** Environmental social sciences, Research data, Geography

## Abstract

Multiple aspects of our society are reflected in how we have transformed land through time. However, limited availability of historical-spatial data at fine granularity have hindered our ability to advance our understanding of the ways in which land was developed over the long-term. Using a proprietary, national housing and property database, which is a result of large-scale, industry-fuelled data harmonization efforts, we created publicly available sequences of gridded surfaces that describe built land use progression in the conterminous United States at fine spatial (i.e., 250 m × 250 m) and temporal resolution (i.e., 1 year - 5 years) between the years 1940 and 2015. There are six land use classes represented in the data product: agricultural, commercial, industrial, residential-owned, residential-income, and recreational facilities, as well as complimentary uncertainty layers informing the users about quantifiable components of data uncertainty. The datasets are part of the Historical Settlement Data Compilation for the U.S. (HISDAC-US) and enable the creation of new knowledge of long-term land use dynamics, opening novel avenues of inquiry across multiple fields of study.

## Abstract (summary)

**Table:** 

Measurement(s)	Structural Land Use
Technology Type(s)	Python
Sample Characteristic - Organism	Structures
Sample Characteristic - Environment	Built Environment
Sample Characteristic - Location	United States

## Background & Summary

Land use, land cover, and settlement databases are typically remote sensing derived or combined products that have made significant contributions to the scientific study of environmental and human systems, but they are limited in their temporal coverage and may suffer from low classification accuracy and limited thematic depth^1–4^. Furthermore, lack of processing infrastructure has created significant obstacles towards advancing our understanding of historical settlement development^5–7^. With increasing data availability and technological advances, large-scale historical-spatial data infrastructures become increasingly feasible and popular in the social and natural sciences^8,9^. As such, data products like the National Land Cover Dataset (NLCD)^1,10,11^ or the Global Human Settlement Layer (GHSL)^12^ typically characterize physical properties of surfaces measured through remotely sensed signals over time but cannot depict thematic details of settlements (e.g., land use classes). No such data exists prior to the 1970s when remote sensing-based earth observation became operational at a global scale. Consequently, researchers are able to evaluate and quantify changes in developed land, the intensity of development, or the proportion of built-up land over a few decades but have a limited understanding of the semantic and functional components of building- and property-related land use and its changes. Furthermore, existing datasets extending farther back in time are typically model-based, of unknown accuracy and of low spatial detail^13,14^.

Significant advancements in our understanding of rural-urban development can only be made if we are able to capture the underlying spatio-temporal processes that contribute to land change at fine scale. However, to date, significant obstacles in alternative data availability and the computational costs of extracting relevant information^15–17^, the low spatial detail contained in historical records^18^ and limited geographic coverage^19–21^ have hindered our ability to produce fine resolution data layers that depict different aspects of land development in urban and rural settings over longer time periods and over large spatial extents.

The multi-temporal land use layers described in this article fundamentally differ from previous instalments of land cover/land use data for its attribute richness, temporal extent and fine temporal and spatial resolution. We detail the creation and properties of a novel gridded data product featuring built up land use progression in the United States from 1940 to 2015. This product was created using the Zillow Transaction and Assessment Dataset (ZTRAX), which is a collection of more than 200 million geocoded housing and property-level records, collected from existing cadastral data sources^22^. These records were rasterized to generate two primary datasets covering the period 1940–2015, a land use majority layer with an annual temporal resolution and class-specific property count layers with a semi-decadal temporal resolution, at a spatial resolution of 250 m for most of the contiguous United States (CONUS), encompassing six different land use classes.

This unique dataset has the potential to transform our understanding of how the compositions of communities and urban centres in the U.S. have developed over 75 years. These data products will be highly useful to researchers in the social and natural sciences, and applicable to studies related to urban development, vulnerability, and natural hazards. While ZTRAX is a proprietary data source, the data derivatives described herein are disseminated as public data to the research community. The historical land use data products are published as part of the Historical Settlement Data Compilation for the U.S. (HISDAC-US)^23,24^ and will be accessible through Harvard Dataverse (https://dataverse.harvard.edu/dataverse/hisdacus). HISDAC-US has been used in several recent studies on urban development and change^25–30^, landscape change analysis and modelling^31^, transportation infrastructure analysis^32^, population modelling^33^, as well as natural hazard risk assessment^34,35^.

## Input Data and Methods

### Semantic aggregation of ZTRAX land use types

The ZTRAX dataset contains information on more than 200 million parcels using over 400 million public records^22^. Third party providers and internal initiatives were used to collect data from assessor information and publicly available documentation. The attribute richness of this dataset offers unique opportunities to explore land use progression and the built environment through novel and compelling perspectives. Recently, ZTRAX has gained increased popularity in the natural and social sciences^36–54^.

The presented land use data product contains six thematic classes of the built environment, which represent land use types of built structures. The six thematic classes described in the data presented herein include: ***agriculture***, ***commercial***, ***industrial***, ***residential-owned***, ***residential-income***, ***and recreational facilities***. The six classes used herein represent a subset of the rich land use classification used in ZTRAX (300 + land use classes total) and were chosen for their importance in studying urban dynamics and development^55–59^. There are 12 general thematic classes contained in ZTRAX; agriculture, commercial, exempt, government, historical, industrial, miscellaneous, private, residential, recreational, transportation, and vacant. Due to the low overall representation and incompleteness of several classes and the importance of the 6 contained in the described data product, 7 of the classes (exempt, government, historical, miscellaneous, private, transportation, and vacant) were omitted from the data and the residential class was subdivided into residential-owned and residential-income. These omissions are reflected in the uncertainty shapefiles and gridded layers we have provided and characterized by the county cumulative sum attribute and grid cell counts. Moreover, we report the subclasses of the included and excluded land use categories and their frequencies^60^.

The ***agricultural*** thematic class in ZTRAX contains 23 subclasses that define agricultural land parcels in greater detail. For our purpose, all non-structural (i.e., not built up) agricultural subclasses were removed from the data prior to processing, keeping all structures such as farms, ranches, miscellaneous structures, and non-residential rural structure improvements. This step ensures that all classes are defined based on built-up structures and not the general use of the land. Examples of excluded agricultural land uses are grazing land, crop land, and other uses that do not describe a physical structure. The reader is directed to the circular histogram^60^ for a complete breakdown of all 300 + land use types and the frequencies in which they appear in the ZTRAX database.

The ***commercial*** sector contains 65 subclasses that range from office and medical buildings to dry cleaners, casinos, and gas stations - no data were removed from this class. For the ***industrial*** theme, 44 subclasses are included in the ZTRAX data that differentiate between heavy industrial buildings such as labour camps, quarries, and slaughterhouses as well as lighter industrial facilities such as assembly plants, recycling centres, and loft buildings. The residential (or housing) sector is broken down into two primary categories 1) ***residential-owned**** (RO)* or residential structures that are owned by a residential account holder who owns the property at the service address of record (https://www.lawinsider.com/dictionary/residential-owner), and 2) ***residential-income**** (RI)* or residential structures that have been zoned as rented or leased dwellings (i.e., not occupied by the owner)^60^. The housing sector contains 36 subclasses that describe residential housing, all of which were included in the final gridded product.

Finally, the ***recreational*** land use class includes recreational facilities that contain 32 subclasses including bowling alleys, playgrounds, zoos, and dance halls. The land use attribute can have three levels of granularity. At the finest granularity, attributes differentiate between aspects of subclasses such as the quality of duplex housing. For the presented data products, we included attributes limited to the primary thematic classes. Table 1 shows the progression of records for these land use classes since 1940 and the circular histogram^60^ shows the sub-classes included in each thematic class represented in the data.

**Table 1:** The cumulative number of ZTRAX property records per land use theme and year.

Land Use Type	1940	1985	2015
Agriculture	170,378	371,886	6,238,359
Commercial	586,298	1,986,790	5,135,934
Industrial	61,256	368,772	938,317
Recreational	11,761	51,729	246,247
Residential-Income	1,656,334	2,980,961	4,117,566
Residential-Owned	11,535,823	51,435,548	100,062,915

### Gridded surface creation

The ZTRAX database is based on cadastral parcel and tax records obtained from state and county records and contains more than 400 million total records^22^, of which approximately 200 million have spatial information. This spatial information typically consists of address points or approximate parcel centroid locations. Due to differing reporting practices from county to county there are swaths of the country that are poorly represented, particularly in the Midwest and Louisiana. According to Zillow’s documentation legal transactions of a house are processed by the county recorder’s office, and it is somewhat common for county recorders to not record the address or assessor parcel number (APN) on the legal records. In such cases it is not possible to systematically map these records to the specific parcels involved (https://www.zillow.com/research/ztrax/ztrax-faqs/). The lack of APN or address manifests as areas of no data (e.g Wisconsin & Louisiana) in the dataset presented in herein.

We converted the ZTRAX data (available as CSV data) into a set of relational databases for efficient querying. We extracted property locations, land use and built year attributes and assigned a bi-dimensional spatial index (i.e., a grid cell identifier) to each record, referenced to a 250 m × 250 m grid in Albers Equal Area Conic projection (SR-ORG:7480) (https://spatialreference.org/ref/sr-org/7480/). This grid is consistent with the spatial grid used in other data products of the HISDAC-US. Then, we separated the data into complete records, and records with missing built year or land use attributes (Section 2.3). We rasterized the complete data records to generate grid cell level land use statistics in annual and semi-decadal cross-sections as defined by a built year attribute of each record^23,24^. Specifically, these records were grouped into spatio-temporal bins (as defined by the grid cell identifier and the built year attribute) and processed to determine the most frequently occurring land use type per grid cell annually from 1940–2015. These summary statistics (i.e., most frequent land use type per grid cell id and year) were then used as input for the rasterization process. We used the Numpy^61^ and Rasterio^62^ Python packages to generate gridded surfaces in GeoTiff format. We also used this process to calculate the counts of each primary land use class per grid cell for each semi-decade starting in 1940. The data with missing spatial references and missing attribute values (i.e., built year, land use type) were then used to calculate various uncertainty statistics using the pandas^63^ and geopandas^64^, and base Python packages^65^. Thus, this data product consists of three items: (a) annual predominant (majority class) land use layers, (b) semi-decadal layers measuring the number of properties of each land use class per grid cell, and (c) accompanying uncertainty surfaces.

### Creation of uncertainty layers

Uncertainty in the created data consists of several components: (a) ZTRAX data incompleteness due to attribute missingness or missing geolocation (i.e., non-georeferenced records), (b) survivorship bias due to historical land use changes not captured by ZTRAX, and (c) thematic uncertainty in the land use attribute. While the latter two types of uncertainty are attempted to be quantified by means of ancillary data (see Section 3), the ZTRAX attribute incompleteness can be quantified directly and is reported in accompanying datasets as additional county-level and grid-cell level summary statistics. Moreover, ZTRAX suffers from positional inaccuracies due to the approximation of areal parcel units by discrete point locations and related issues^50^. In earlier work, we quantified and reported positional, thematic and temporal uncertainties in existing settlement layers and provided uncertainty layers hosted in the HISDAC-US repository^23,24^ (https://dataverse.harvard.edu/dataverse/hisdacus). These uncertainty layers are highly recommended for users interested in applying the historical settlement data products. The uncertainty layers described here focus on data missingness in creating time sequences of gridded land use layers at the county-level and at the grid cell-level.

We calculated the proportion of records with missing land use attributes and/or missing geolocation to quantify the ***county-level uncertainty***. We determined the total count of records in each county and calculated the proportion of records with and without a georeference. Moreover, we cross-tabulated attribute missingness for the built year (“by”) and land use (“lu”) attributes:

1. proportion of records that contained both a land use and year-built value (“by-lu”)
2. proportion of records without both land use and year-built values (“nby-nlu”)
3. proportion of records with land use and no built year (“nby-lu”)
4. proportion of records with a built year and no land use (“by-nlu”)

In order to further characterize the county-level attribute missingness over time, we generated decadal, county-level shapefiles containing the proportion of records with valid year-built attribute, but missing land use attribute per county. This resulted in seven county boundary files, one for each decade within the temporal coverage of our data. For each decade we then calculated proportions of georeferenced records, proportions of records that had both the built year and land use attribute, as well as the proportions of records with missing land use attribute for that decade. Additionally, we created ***grid cell-level uncertainty layers***. To gain a fine-resolution understanding of uncertainty, we generated gridded uncertainty layers using the georeferenced records in the ZTRAX data. Gridded time sequences (decadal) were created using all the georeferenced records that had a value for the built year but no land use attribute to quantify the proportion of missing land use type entries at the grid cell level. These uncertainty layers are recommended for data users to integrate in their analysis to be able to account for varying data quality, both regionally and over time.

## Data Records

### Historical gridded land use layers

The datasets described in the following sections have been published in the Harvard Dataverse HISDAC-US repository at the following URL https://dataverse.harvard.edu/dataverse/hisdacus^66–68^. The multi-temporal land use surfaces are organized as sequences of georeferenced gridded layers (file names include the year e.g., LU_ThemeMaj_1985) covering most of the built-up areas in CONUS (excluding Hawaii, Alaska, and non-covered counties) with a spatial resolution of 250 m and a temporal resolution of one year for the majority class data product, and 5 years for the class-specific layers. In the main data product, each grid cell value represents the most frequently occurring land use class among all ZTRAX records located within that grid cell, for a given year (for all georeferenced records with both a built year and a land use designation). Additionally, for each individual land use class, we created a time sequence of gridded count layers representing the number of records of that land use class (e.g., industrial) located within a grid cell for each semi- decade starting in 1940. These layers have the land use class and the year included in their file names (e.g., LU_ThemeCount_RO_1975, for residential-owned structures in 1975). These data products cover the time period 1940–2015. We have provided the raster layers in GeoTIFF format with a spatial resolution of 250 m. We aligned these layers to the existing layers in the HISDAC-US to ensure consistency across settlement data products housed in that data compilation. We have published all data in the HISDAC-US repository using the Albers Equal Area Conic projection for the contiguous US (USGS version, SR-ORG:7480).

**Fig. 1:**
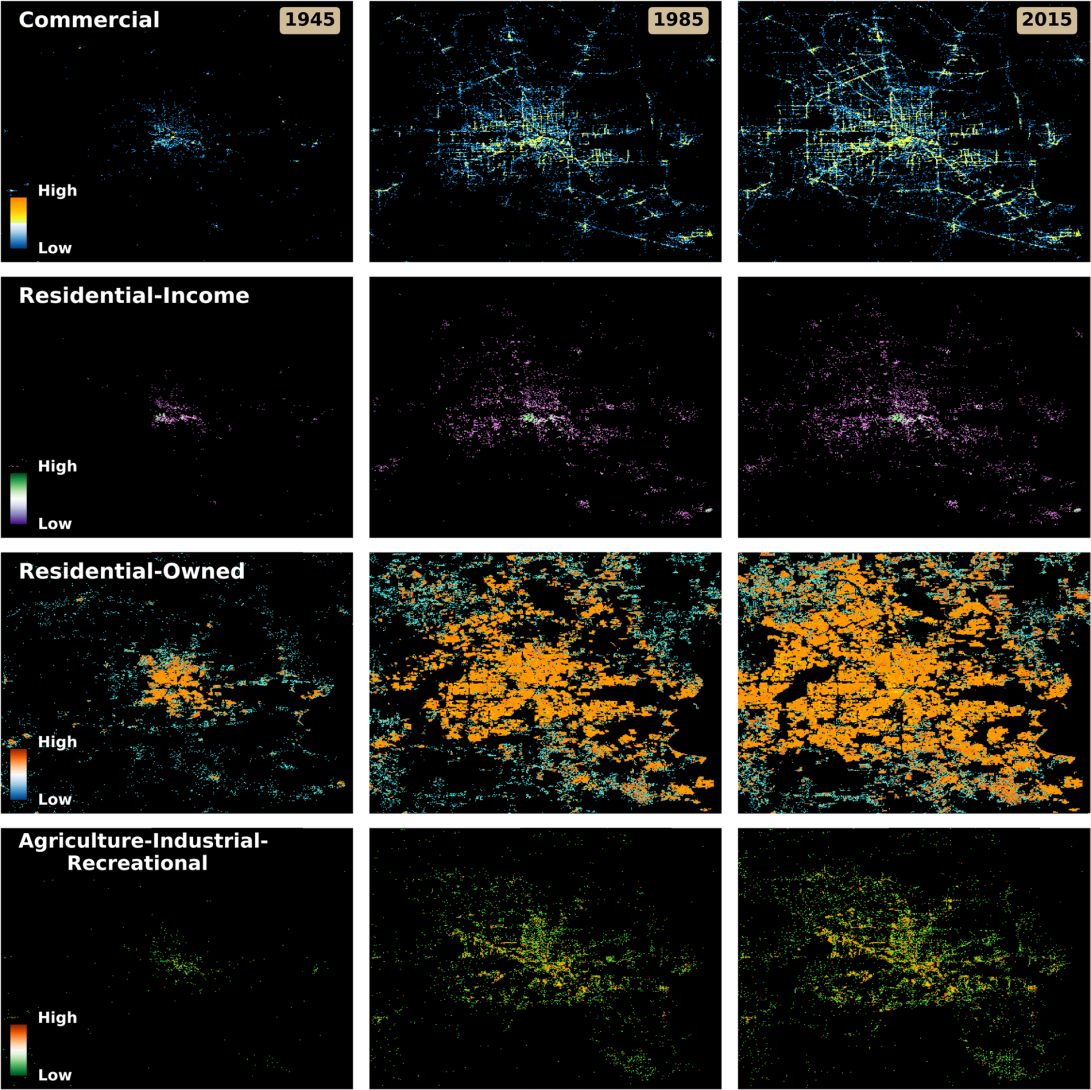
Land use-specific property counts in 1945, 1985, and 2015, for Houston, Texas. The top 3 rows display theme specific counts. The bottom row displays the aggregated counts of the agricultural, industrial, and recreational land use classes.

Figure 1 illustrates various aspects of the land use data package that offer novel perspectives on urban development. The dataset allows the user to understand urban growth in terms of land use change, not only through thematic majority but count surfaces that characterize growth of land use classes over time. The top 3 rows in Fig. 1 show the cumulative counts for commercial, residential-income, and residential-owned land use classes, at three points in time in Houston, Texas. The bottom row in Fig. 1. displays the cumulative counts for all other land uses classes, Agriculture, Industrial, and Recreational. Additionally, we have generated contemporary (i.e., 2016) count surfaces for the thematic classes and accompanying uncertainty surfaces. These 2016 layers also contain those records that lack a built year record and thus represent a more complete picture of more recent land use patterns.

### Uncertainty surfaces

As described above, *w*e have created several uncertainty surfaces in order to provide information on basic data quality aspects. Data completeness and multi-variable processing quickly creates a complex picture of uncertainty. There are two categories of uncertainty layers that we have provided: vector files with multi-temporal data in the attribute table aggregated to the county level (2010-boundaries) (https://www.census.gov/geographies/mapping-files/time-series/geo/carto-boundary-file.html) and multi-temporal gridded uncertainty layers consistent with the main data products.

Seven county-boundary vector layers have been provided, one for each decade. Each layer provides the proportion of records that are georeferenced and the proportion of records that are not georeferenced but located in that county based on the county identifier, for each decade. Additionally, we have provided the proportion of records that have a built year and the proportion of records that have a land use attribute for that county and year for both the georeferenced and non- georeferenced data. Due to the exclusion of poorly represented land use types (i.e. exempt, government, historical buildings) there are counties in the uncertainty surfaces that have more cumulative structures listed in the year-built attribute than listed in the land use attribute column. The instances in which there are more structures for a given year than counted in the land use attribute represent structures with land use types that were omitted from the land use data. We provided an additional decadal gridded uncertainty layer to address the structures that were excluded from the dataset. For each decade we calculated the cumulative number of excluded structures per grid cell. There are 6 thematic classes excluded from the main data product and 12 agricultural sub-classes that were excluded as they did not characterize built up structures. The 6 non-agricultural thematic classes represented by this gridded uncertainty layer are: (1) Exempt, (2) Historical, (3) Miscellaneous, (4) Privately Owned, (5) Transportation, and (6) Vacant. The data user is urged to use those layers, and the detailed land use disaggregation^60^ to inform their analysis using baseline data qualities and completeness information.

The multi-temporal gridded uncertainty layers for all georeferenced data quantify missingness in land use type entries at the same resolution as the main data product. Each surface represents only those structures that were explicitly geocoded in ZTRAX. There are five attributes that characterize uncertainty in the county level shapefiles: (1) the cumulative sum of all structures contained in ZTRAX for each county and decade, (2) the cumulative sum of all structures containing a land use attribute per county and decade, (3) the cumulative sum of all structures with a built year attribute per county and decade, (4) the proportion of structures containing the land use attribute relative to all structures in the county per decade, and finally (5) the proportion of structures containing the year built attribute relative to all structures in the county per decade. The shapefile variables containing proportions represent the completeness of either the land use or built year attributes. Data users are encouraged to use the uncertainty surfaces provided with the data presented herein and the positional uncertainty layers published in HISDAC-US^23,24^ to assess data suitability for a given location and to account for inherent positional uncertainty. Below we provide a table (Table 2) describing the files contained in the land use data sets.

**Table 2:** Technical specifications and access information for the created historical land use datasets.

File name	Description	Temporal resolution	Temporal coverage	Spatial resolution	File Format	URL	DOI
LU_ThemeMaj_YYY Y.tif	Annual gridded surfaces depicting the majority land use class per grid cell	1 year	1940–2015	250 m × 250 m	GeoTIFF	https://dataverse.harvard.edu/dataset.xhtml?persistentId=doi:10.7910/DVN/LNBJIO	10.7910/DVN/LNBJIO
LU_ThemeCount_A_YYYY_to_YYYY.tif	Semi decadal gridded surface showing the cumulative count of agricultural structures	5 years	1940–2015	250 m × 250 m	GeoTIFF	https://dataverse.harvard.edu/dataset.xhtml?persistentId=doi:10.7910/DVN/I30REZ	10.7910/DVN/I30REZ
LU_ThemeCount_C_YYYY_to_YYYY.tif	Semi-decadal gridded surface showing the cumulative count of commercial structures	5 years	1940–2015	250 m × 250 m	GeoTIFF	https://dataverse.harvard.edu/dataset.xhtml?persistentId=doi:10.7910/DVN/I30REZ	10.7910/DVN/I30REZ
LU_ThemeCount_I_YYYY_to_YYYY.tif	Semi-decadal gridded surface showing the cumulative count of industrial structures	5 years	1940–2015	250 m × 250 m	GeoTIFF	https://dataverse.harvard.edu/dataset.xhtml?persistentId=doi:10.7910/DVN/I30REZ	10.7910/DVN/I30REZ
LU_ThemeCount_R C_YYYY_to_YYYY.tif	Semi-decadal gridded surface showing the cumulative count of recreational structures	5 years	1940–2015	250 m × 250 m	GeoTIFF	https://dataverse.harvard.edu/dataset.xhtml?persistentId=doi:10.7910/DVN/I30REZ	10.7910/DVN/I30REZ
LU_ThemeCount_R I_YYYY_to_YYYY.tif	Semi-decadal gridded surface showing the cumulative count of residential-income structures	5 years	1940–2015	250 m × 250 m	GeoTIFF	https://dataverse.harvard.edu/dataset.xhtml?persistentId=doi:10.7910/DVN/I30REZ	10.7910/DVN/I30REZ
LU_ThemeCount_R O_YYYY_to_YYYY.tif	Semi-decadal gridded surface showing the cumulative count of residential-owned structures	5 years	1940–2015	250 m × 250 m	GeoTIFF	https://dataverse.harvard.edu/dataset.xhtml?persistentId=doi:10.7910/DVN/I30RE	10.7910/DVN/I30REZ
LuUncert_County_ YYYY _to_YYYY.shp	Decadal shapefile surfaces describing the attribute missingness for land use and built year for all records	10 years	1940–2015	County	ESRI Shapefile	https://dataverse.harvard.edu/dataset.xhtml?persistentId=doi:10.7910/DVN/JXJ5WH	10.7910/DVN/JXJ5WH
LuUncert_County_ 2016.shp	Shapefile surface that describes the attribute missingness using all records missing one or both (land use, built year) attributes	—	1940–2015	County	ESRI Shapefile	https://dataverse.harvard.edu/dataset.xhtml?persistentId=doi:10.7910/DVN/JXJ5WH	10.7910/DVN/JXJ5WH
LU_UncertPix_YYYY _to_YYYY.tif	Decadal gridded surfaces describing the land use attribute missingness for all georeferenced records	10 years	1940–2015	250 m × 250 m	GeoTIFF	https://dataverse.harvard.edu/dataset.xhtml?persistentId=doi:10.7910/DVN/JXJ5WH	10.7910/DVN/JXJ5WH
LU_UncertPix_2016 s.tif	Gridded surface showing the attribute missingness for both land use and built year	—	2015	250 m × 250 m	GeoTIFF	https://dataverse.harvard.edu/dataset.xhtml?persistentId=doi:10.7910/DVN/JXJ5WH	10.7910/DVN/JXJ5WH
Uncert_ExcldLU_YYYY_to_YYYY.tif	Gridded surface showing cumulative counts of structures represented in ZTRAX and excluded from the land use data	10 years	1940–2015	250 × 250	GeoTIFF	https://dataverse.harvard.edu/dataset.xhtml?persistentId=doi:10.7910/DVN/JXJ5WH	10.7910/DVN/JXJ5WH

## Technical Validation

ZTRAX is subjected to quality issues that include spatial, temporal, and thematic uncertainties that propagate into the gridded surfaces contained in the HISDAC-US. In part, these uncertainties have been quantified in previous work^23,24^. For a thorough positional accuracy assessment of the gridded surfaces in HISDAC-US, over time and across the rural-urban continuum, we direct the reader to Uhl *et al*. (2021a), who report regionally varying levels of positional agreement. Uhl *et al*. (2021a) provide important insights into the quantity agreement of the ZTRAX-derived grid cell aggregates of built-up records and locations, as compared to building footprint data, census population and housing unit counts. Such reported disagreements also propagate into the land use data layers described herein, and the user of any data products from the HISDAC-US is urged to refer to these validation results to reflect the accuracy of the data appropriately. Based on this validation, it is known that while the completeness in HISDAC-US is acceptable for data layers after 1900, all products derived from ZTRAX will be subject to underestimation due to the difficulties of obtaining structural records from counties that have differing reporting policies, attribute incompleteness and inconsistency, and the dynamic nature of development. The level of underestimation for residential records was assessed in Uhl *et al*. (2021a) who reported varying levels of incompleteness of records along the rural-urban continuum in comparison to Census housing unit counts.

Herein, we assessed the completeness of land use and year-built attributes in ZTRAX (Section 4.1) and employed three ancillary datasets to quantitatively and qualitatively address uncertainties specific to the land use product. Specifically, we used land use data from volunteered geographic information (i.e., OpenStreetMap, OSM) (https://planet.openstreetmap.org) to assess the agreement with the created (contemporary) land use layers (Section 4.2), and compared our land use layers to remote-sensing-derived land cover/land use (LULC) data from the National Land Cover Database 2001^69^ and 2016^70^, as well as to urban land use classes from the Local Climate Zones (LCZ)^71^ dataset available for the CONUS (Section 4.3). In addition to that, we used data on building demolitions to quantify effects of survivorship bias, as building replacements or teardowns are not recorded in ZTRAX (Section 4.4). Finally, we used overhead imagery and a visual-analytical approach to assess the visual consistency of buildings at ZTRAX locations for different land use categories (Section 4.5).

### Attribute incompleteness

**Fig. 2:**
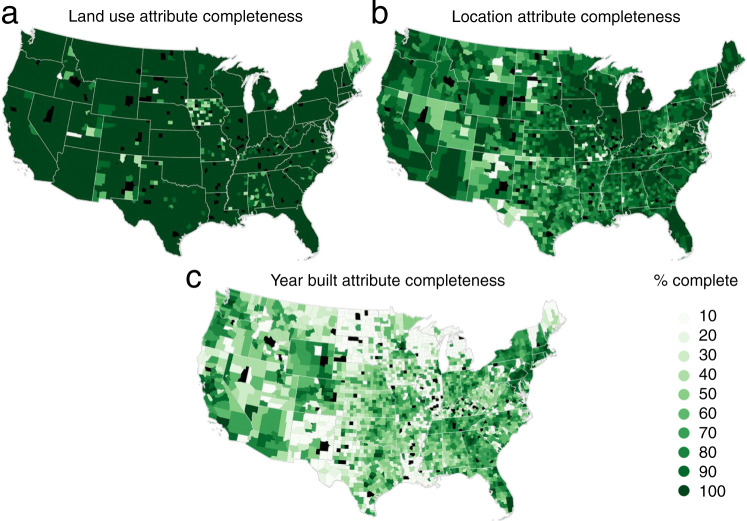
Attribute completeness in ZTRAX: Percentage of records per county with a valid (**a**) land use attribute, (**b**) location attribute (i.e., latitude and longitude), and (**c**) year built attribute.

Grid cells with small structural counts and low attribute completeness should be carefully considered as cell value assignment in the main data product was based on the most frequently occurring land use class within the grid cell extent. In such cases, the user is advised to use the land use type count layers in conjunction with uncertainty layers to better understand the underlying reliability of the data. Table 3 summarizes attribute missingness statistics for the land use data product, indicating that over 98% of the ZTRAX records have valid land use information (lower levels are found e.g. in Maine or Iowa, see Fig. 2a), and over 75% of ZTRAX records have valid land use and year built information. The majority of ZTRAX records have valid location information (Fig. 2b). Around 25% of the data are lacking land use and year-built information, and these are located in approximately 400 counties (see Fig. 2c), that can be also identified by the county-level completeness layers (Section 3.2).

**Table 3:** Cross-tabulation of land use (lu) and year built (by) completeness; “n” indicates missingness, e.g., nby = “no built year”.

	Counts [N]			Percentages [%]		
	nby	by	sum	nby	by	sum
nlu	2187645	77796	2265441	1.76	0.06	1.82
lu	28744633	93272917	1.22E + 08	23.13	75.05	98.18
sum	30932278	93350713	1.24E + 08	24.9	75.11	100

### Comparison to OpenStreetMap land use data

While detailed and reliable land use data is sparse, OpenStreetMap (OSM) offers user-generated land use and functional information at the building level. While OSM is not expected to have high completeness in terms of the land use attribute, we assume the reported land use information to be accurate. We generated gridded surfaces, aligned with the HISDAC-US land use data grids, containing the number of buildings of a given land use type in OSM per grid cell, and conducted a cell-level agreement assessment. We mapped the relevant OSM land use types to the land use classification scheme of the presented HISDAC-US land use data. Moreover, due to the sparsity of some land use classes in both datasets, and the potentially large bias introduced by this, we only evaluated the three most frequent land use classes: residential, commercial, and industrial.

Preliminary tests have shown that a considerable amount of building footprints in OSM are lacking the land use attribute and thus, its completeness in OSM appears to be low in certain regions of the CONUS, while the correctness of those attributes that exist is expected to be high. Thus, only Type II errors (i.e., comission errors) in the ZTRAX-derived land use data can be quantified by comparing against the OSM data. For the evaluation of commission error, please refer to Section 4.3 (comparison to remotely-sensed LULC data) and Section 4.4 (Survivorship bias). Note that this assessment was done for the most recent point in time of the presented land use layer series (i.e., 2016) and for contemporary OSM data downloaded in 2021, to keep the temporal gap to a minimum. We carried out these assessments for individual counties as well as across the rural-urban continuum using the Rural-Urban Continuum Codes (RUCCs) created by the U.S. Department of Agriculture (USDA)^72,73^. RUCCs define nine rural-urban classes, including three metro and six non-metropolitan county designations using criteria of population size, the degree of urbanization and adjacency to a metro area (Table 4).

**Table 4:** OSM-based agreement assessment using correlations and recall measures across the rural-urban continuum (RUC).

RUCC	Spearman correlation			Recall		
	Residential	Commercial	Industrial	Residential	Commercial	Industrial
**1** (**urban**)	0.663	0.368	0.585	0.883	0.652	0.432
Pop > = 1 m						
**2**	0.621	0.254	0.26	0.707	0.576	0.233
Pop > = 250 K & pop <1 m						
**3**	0.601	0.292	0.261	0.569	0.534	0.195
Pop <250 K						
**4**	0.652	0.296	0.15	0.696	0.526	0.194
Pop> = 20 K adjacent to metro area						
**5**	0.575	0.252	0.162	0.682	0.579	0.148
Pop > = 20 K & not adjacent to metro area						
**6**	0.557	0.297	0.31	0.504	0.437	0.103
Pop > = 2,500 & pop < = 19,999 adjacent to metro						
**7**	0.586	0.284	0.033	0.339	0.347	0.062
Pop > = 2,500 & pop < = 19,999 not adjacent to metro						
**8**	0.494	0.337	−0.065	0.36	0.344	0.047
Pop < = 2,500 adjacent to metro area						
**9** (**rural**)	0.523	0.184	−0.087	0.337	0.256	0.039
Pop < = 2,500 not adjacent to metro area						
CONUS	0.676	0.334	0.546	0.77	0.608	0.336

Brief descriptions of each RUCC are provided in terms of population (pop) below the RUCC designation (1 = urban, 9 = rural).

**Fig. 3:**
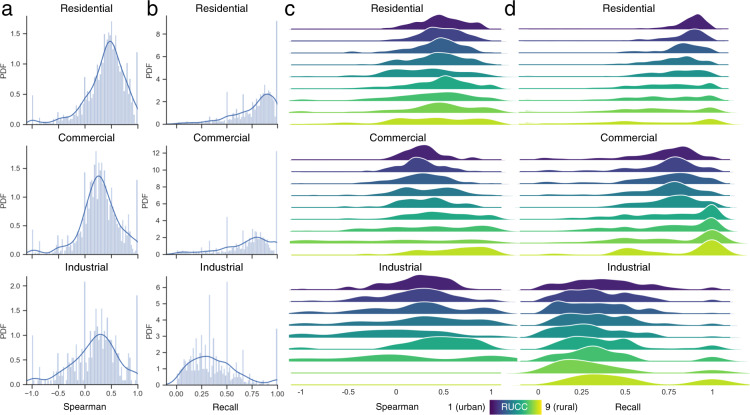
Comparison to building-level land use classes from OpenStreetMap. (**a**) Distribution of Spearman’s correlation coefficient based on 250 × 250 m grid cell counts of residential, commercial, and industrial records, and (**b**) distribution of county-level recall values; Panels (**c**) and (**d**) show the distributions of county-level correlation and recall, disaggregated for each rural-urban continuum code.

Given these constraints in the OSM reference data, we first extracted all grid cells containing at least one OSM and ZTRAX derived record of the same land use class and assessed the correlations of the grid cell counts, as a measure of quantity agreement. Due to the ZTRAX data structure and spatial generalization effects, these distributions can contain outliers, resulting from large numbers of records in individual grid cells^24^, and thus, we used Spearman’s rank correlation coefficient for this assessment. Moreover, we calculated the recall (i.e., producer’s accuracy, or sensitivity) of the ZTRAX-derived land use counts with respect to the OSM in order to quantify the omission errors associated with the ZTRAX-derived data. The latter was done based on binarized absence-presence gridded surfaces, using a threshold of at least one record per grid cell, and thus allowing for measuring the Type II error component of the positional agreement between the ZTRAX and OSM derived surfaces. We quantified both, correlations of grid-cell level counts and the spatial agreement (i.e., recall) for each of the three land use classes under test for the whole CONUS, and across the rural-urban continuum, by conducting stratified assessments for grid cells located in counties of each RUCC (Table 4), as well as for each individual county (Fig. 3).

First, we observe positive correlations between building counts of ZTRAX and OSM land use classes across CONUS (>0.33 across all RUCCs for any class), and these correlations are highest for the residential class in highly urban environments (i.e., RUCC 1, c = 0.66). Correlations generally decrease towards more rural settings, where both ZTRAX and OSM completeness can be low. The completeness of ZTRAX land use records appears to decrease from the residential to the commercial and industrial classes, yielding recall values over all RUCCs of up to 0.77, 0.61 and 0.34, respectively. Recall values across the RUC follow similar patterns as the correlations, exhibiting highest values for the residential class in urban settings (RUCC 1, recall = 0.88) and lowest values in rural settings for the industrial class.

While these general patterns illustrate the broad-scale agreement between ZTRAX and OSM based land use data, we observe strong local variations of uncertainty at the county level, as the distributions of Spearman’s rank correlation coefficients and recall measures calculated at the county level suggest (Fig. 3a,b). As the upper tails of these distributions indicate, there is a considerable number of counties that exhibit very high quantity and positional Type II agreement between ZTRAX and OSM. Decomposing the distributions of county-level agreement metrics across the rural-urban continuum, we observe that while the overall agreement metrics in Table 4 decrease from urban towards rural regions, this trend is less visible in the county-level metrics (Fig. 3c,d). For example, a considerable number of rural counties (RUCC 6–9) exhibit high recall values for residential and commercial land use classes. We would like to emphasize that different factors such as spatial, temporal, and semantic inconsistencies between ZTRAX and OSM data, as well as the user-generated nature of the OSM database and associated uncertainty issues affect the presented agreement assessment results, underlining the difficulty in conducting land use data validation in general. However, these results suggest that the surfaces representing contemporary land use are largely coherent to the independently collected and compiled OSM data and thus, represent a reliable and plausible proxy for land use distributions in most regions of the CONUS.

#### Comparison to remote-sensing-based LULC datasets

We used gridded land cover data from the NLCD in 2001^69^ and 2016^70^, as well as gridded LCZ urban land use (temporally referenced approximately in 2016–2018) available for the CONUS^71^. We implemented two approaches for this comparison: First, we implemented a **record-based approach**: We drew a stratified random sample of ZTRAX records, retrieved the land use/climate zone from the underlying NLCD and LCZ grids at the location of each record, and cross-tabulated the land use class of each ZTRAX record and the respective NLCD and LCZ class labels found at the respective location. Specifically, we randomly selected one county for each of the nine RUCCs, within each of the nine U.S. census divisions^72,73^. We then retrieved the ZTRAX property records within these counties and drew a sample of n = 1,000 records (with replacement) from each of our six land use classes (cf. Table 1) per county. This way, we obtained a sample of N = 486,000 ZTRAX records, located within 81 U.S. counties uniformly distributed across the CONUS, and across the rural-urban continuum, and equally proportioned across the land use classes used herein.

Second, we implemented a **raster-based approach**: We down-sampled the NLCD gridded surfaces from their native resolution of 30 m and the LCZ data from 100 m into the HISDAC 250 m grid using two resampling techniques: 1) a majority-area rule, and 2) using 1-hot encoding, i.e., creating a binary 250 m gridded surface for each NLCD and LCZ class, encoding the presence of each class with 1, and the absence with 0. This way, we were able to evaluate the correspondence of our land use classes also to underrepresented classes in NLCD and LCZ, which are likely to disappear when using majority-area resampling. Similarly, we created a binary surface in our 250 m grid indicating the presence (1) or absence (0) of records of any of our six land use classes, based on the land-use-specific property count surfaces (cf. Fig. 2). We compared these data layers by cross-tabulating our land use based binary surface with the binary surfaces of each of the LULC classes from NLCD and LCZ, respectively.

We compared NLCD 2016 and LCZ to our 2016 layers, and to minimize the effects of temporal inconsistencies, we compared the NLCD 2001 to our layers referenced in the year 2000. These different strategies (record-based and raster-based) allowed for gaining a relatively unbiased picture of the correspondence between our land use classes and remotely sensed LULC types. The record-based approach evaluates the correspondence between ZTRAX and the LULC datasets without being affected by additional uncertainty induced by the resampling. However, it only evaluates thematic agreement where ZTRAX records are available, disregarding omission errors. The raster-based approach may suffer from additional positional uncertainty due to resampling from 30 m (NLCD) and 100 m (LCZ) resolutions to the target resolution of 250 m but enables the quantification of class-specific omission errors in regions where no ZTRAX records are available.

**Fig. 4:**
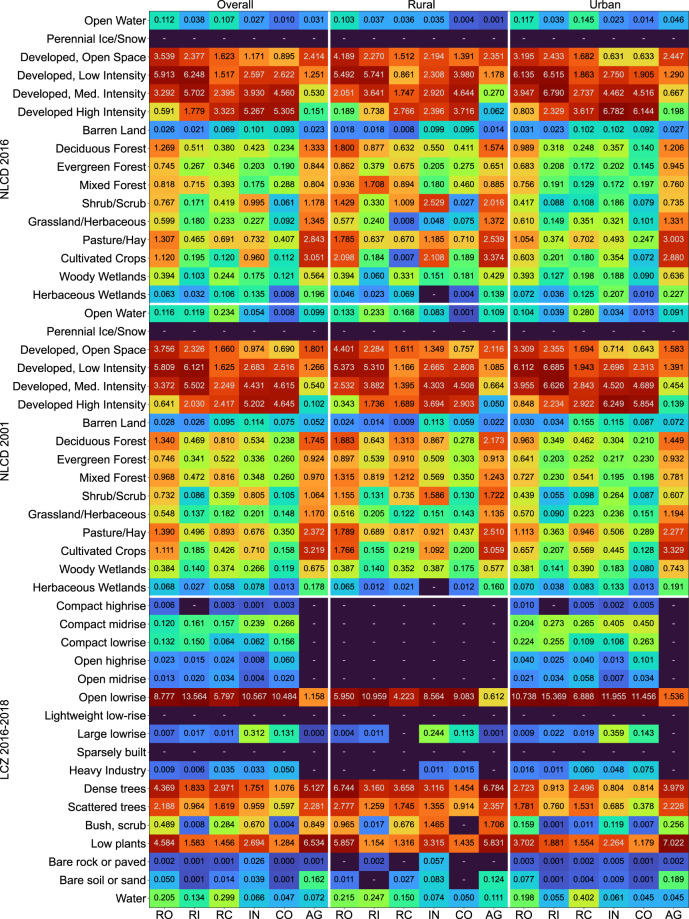
Record-level comparison of ZTRAX land use classes and LULC land use categories, carried out for the full sample, for rural counties (RUCC 6–9) and urban counties (RUCC 1–5). Values are shown in % of the sample of N = 486,000 ZTRAX records used.

The record-based comparison (Fig. 4, top part) revealed very similar patterns for NLCD 2001 and NLCD 2016: Highest proportions of income and owned residential ZTRAX records are located in the NLCD classes “Developed, low intensity” and “Developed, medium intensity”, whereas industrial and commercial land uses have highest proportions in “Developed, high intensity”, in particular in urban counties. Agriculturally used properties have highest proportions within “Pasture/Hay” and “Cultivated crops”. Comparing to the local climate zones (Fig. 4, bottom part) shows that the highest proportions of ZTRAX records for most land use classes are located within the “Open low-rise” class, except for the agricultural land use class, which peaks in the “Low plants” and “Dense trees” LCZ classes.

Some of these cross-tabulations seem implausible, such as ZTRAX records located in wetlands or open water. It is likely that these are artefacts due to the resampling, and the spatial resolution of the HISDAC-US land use data layers. In the least optimistic scenario, we can consider these mismatches to be commission errors (i.e., ZTRAX reports built-up structures that do not exist). In that case, these commission errors quantifiable by the conducted cross-comparison would sum up to only 4–5% of all ZTRAX records. Here, it is worth noting that commission errors in ZTRAX may also occur due to demolished buildings that have not been deleted or updated (i.e., set to “vacant” land use) in ZTRAX. However, these cases are likely not to exceed 1–2% of all ZTRAX records (see Section 4.4).

The raster-based approach reveals a complementary picture. As shown in Table 5, for most non-settlement and vegetation-dominated NLCD classes, only small area proportions are covered by grid cells containing one or more ZTRAX records. This trend inverts for the settlement-related land cover classes: For example, over 82% of “Developed, low intensity” land cover in 2016 geographically coincides with the land use data described herein. This agreement is lower for the NLCD 2001 data, as grid cells without temporal information are counted as “% not covered by HISDAC”. This trend persists across the two different data resampling techniques. Larger differences in these proportions between majority-based resampling and 1-hot encoding indicate that the land cover classes (e.g., “Developed, low intensity”) are underrepresented and/or spatially scattered and thus, disappear when using majority-based resampling. Moreover, when distinguishing these cross-tabulations in proportions of the HISDAC-covered and not covered area (Table 5, bottom part), we observed that the highest proportion of not covered area is shrubland/scrub (i.e., 23%), and highest proportions of the HISDAC-covered areas are located in “Deciduous Forest” and “Pasture/Hay” (agricultural class, cf. Figure 4), followed by the developed classes.

**Table 5:** Grid-cell-level comparison of ZTRAX land use classes and NLCD 2001 and 2016 land cover classes.

	Mode-based resampling				1-hot encoding			
	NLCD 2001		NLCD 2016		NLCD 2001		NLCD 2016	
	not covered by HISDAC	covered by HISDAC	not covered by HISDAC	covered by HISDAC	not covered by HISDAC	covered by HISDAC	not covered by HISDAC	covered by HISDAC
Reference	Proportions of NLCD class							
Open Water	98.18	1.82	97.26	2.74	98.11	1.89	97.17	2.83
Perennial Ice/Snow	100	0	100	0	100	0	100	0
Developed. Open Space	44.67	55.33	32.96	67.04	66.21	33.79	55.94	44.06
Developed. Low Intensity	29.27	70.73	17.8	82.2	47.21	52.79	35.86	64.14
Developed. Medium Intensity	28.12	71.88	20.22	79.78	35.09	64.91	28.16	71.84
Developed High Intensity	36.57	63.43	28.21	71.79	37.96	62.04	30.47	69.53
Barren Land	98.86	1.14	98.47	1.53	98.55	1.45	98.13	1.87
Deciduous Forest	89.6	10.4	84.44	15.56	89.67	10.33	84.65	15.35
Evergreen Forest	96.7	3.3	95.03	4.97	96.65	3.35	95.02	4.98
Mixed Forest	88.86	11.14	83.12	16.88	88.91	11.09	83.44	16.56
Shrub/Scrub	98.95	1.05	98.38	1.62	98.93	1.07	98.35	1.65
Grassland/Herbaceous	98.02	1.98	96.95	3.05	98.09	1.91	97.02	2.98
Pasture/Hay	84.07	15.93	76.01	23.99	85.3	14.7	77.89	22.11
Cultivated Crops	95.35	4.65	91.13	8.87	95.34	4.66	91.27	8.73
Woody Wetlands	95.01	4.99	91.3	8.7	94.69	5.31	90.96	9.04
Emergent Herbaceous Wetlands	97.48	2.52	95.66	4.34	97.03	2.97	94.98	5.02
Reference	Proportions of HISDAC class							
Open Water	5.57	0.1	5.5	0.15	5.52	0.11	5.52	0.11
Perennial Ice/Snow	0.01	0	0.01	0	0.01	0	0.01	0
Developed. Open Space	0.67	0.84	0.52	1.07	1.9	0.97	1.9	0.97
Developed. Low Intensity	0.36	0.87	0.24	1.09	0.81	0.9	0.81	0.9
Developed. Medium Intensity	0.2	0.51	0.18	0.72	0.29	0.54	0.29	0.54
Developed High Intensity	0.1	0.17	0.09	0.23	0.11	0.19	0.11	0.19
Barren Land	1.04	0.01	1.04	0.02	1.06	0.02	1.06	0.02
Deciduous Forest	9.81	1.14	8.92	1.64	9.21	1.06	9.21	1.06
Evergreen Forest	12.87	0.44	12.2	0.64	12.35	0.43	12.35	0.43
Mixed Forest	2.9	0.36	2.67	0.54	3.27	0.41	3.27	0.41
Shrub/Scrub	23.29	0.25	23.19	0.38	22.95	0.25	22.95	0.25
Grassland/Herbaceous	13.98	0.28	14.25	0.45	14.07	0.27	14.07	0.27
Pasture/Hay	6.51	1.23	5.37	1.7	6.26	1.08	6.26	1.08
Cultivated Crops	16.78	0.82	16.68	1.62	16.05	0.78	16.05	0.78
Woody Wetlands	4.51	0.24	4.36	0.42	4.54	0.25	4.54	0.25
Emergent Herbaceous Wetlands	1.39	0.04	1.36	0.06	1.59	0.05	1.59	0.05

The raster-based cross-tabulations with LCZ classes show a similar pattern: The majority of the settlement-related and built-up classes (e.g., compact and open high-rise, etc.) are covered by HISDAC-US (Table 6, left part), whereas most vegetation-dominated LCZ classes are not covered. However, we observed some exceptions deviating from this trend: For example, the “Open midrise” class is mostly not covered in HISDAC-US. A reason could be public buildings that are omitted in HISDAC-US^24^ and are not considered in the land use data presented herein. Moreover, only 10–12% of the grid cells labelled as “Heavy industry” are covered in HISDAC-US. This may be caused by spatial offsets, as industrially used parcels may be very large, but also indicates a relatively poor coverage of industrial land use, which is also in line with observations made when comparing our data to OSM (Section 4.2). Conversely, the highest proportions of HISDAC-US-covered area in LCZ is classified as “Open low-rise”, “Dense trees” or “Low plants”. This is plausible as dense, urban settlements only represent a small portion of the US built environment, and peri-urban and rural settlements as well as agriculturally used structures are typically spatially scattered and thus, as a result of the resampling process, “occupy” a larger proportion of grid cells than built-up properties in dense, urban settings.

**Table 6:** Grid-cell-level comparison of ZTRAX land use classes and LCZ 2016–2018 urban land use categories.

	% of LCZ class				% of HISDAC class			
	Mode-based resampling		1-hot encoding		Mode-based resampling		1-hot encoding	
	not covered by HISDAC	covered by HISDAC	not covered by HISDAC	covered by HISDAC	not covered by HISDAC	covered by HISDAC	not covered by HISDAC	covered by HISDAC
Compact highrise	43.98	56.02	66.29	33.71	0	0	0	0
Compact midrise	11.57	88.43	17.07	82.93	0	0.01	0	0.01
Compact lowrise	0.41	99.59	1.32	98.68	0	0	0	0.01
Open highrise	21.68	78.32	34.11	65.89	0	0	0	0
Open midrise	64.99	35.01	67.93	32.07	0	0	0.01	0
Open lowrise	27.42	72.58	35.96	64.04	1.12	2.97	1.98	3.53
Lightweight low-rise	0	0	0	0	0	0	0	0
Large lowrise	38.15	61.85	41.51	58.49	0.03	0.05	0.06	0.08
Sparsely built	0	0	0	0	0	0	0	0
Heavy Industry	89.09	10.91	87.71	12.29	0.01	0	0.03	0
Dense trees	87.77	12.23	85.54	14.46	22.78	3.17	28.6	4.83
Scattered trees	91.92	8.08	87.96	12.04	15.7	1.38	28.41	3.89
Bush. scrub	99.01	0.99	98.75	1.25	15.1	0.15	20.01	0.25
Low plants	89.82	10.18	88.21	11.79	29.74	3.37	37.83	5.05
Bare rock or paved	99.04	0.96	98.54	1.46	1.39	0.01	2.04	0.03
Bare soil or sand	99.34	0.66	99.02	0.98	10.2	0.07	14.2	0.14
Water	97.49	2.51	93.01	6.99	3.92	0.1	5.75	0.43

It is worth noting that due to the different properties of the data compared herein (i.e., discrete locations vs. categorical and density information contained in gridded surfaces), positional uncertainty in both the LULC data (e.g., induced by the registration accuracy of the underlying remote sensing data) and in the ZTRAX data (e.g., using parcel centroids or address points instead of the locations of actual built-up structures) may introduce additional uncertainty in these cross-comparisons. However, the aggregation of the NLCD and LCZ datasets from fine resolutions to the target resolution of 250 m is assumed to mitigate such bias partially.

### Assessing survivorship bias in the historical data

Survivorship bias presents a problem that appears in several disciplines and is of particular importance to most types of settlement, land use, or building stock data^74–78^. This type of bias appears when units, such as a built structure, are removed from the population but are not accounted for in the data. For example, a structure built in 1930 may get remodelled or may get demolished over time^79^. ZTRAX does not directly account for demolished structures and therefore does not continue to represent structures that no longer exist. The described land use data product suffers from the same limitation in that only *surviving* buildings are considered without accounting for possible structural losses. To demonstrate and measure the effects of this survivorship bias for Colorado, we used address-level demolition data over 10 years (2008–2017) obtained from the Colorado State archives (https://spl.cde.state.co.us/artemis/heserials/he171017internet/).

We stratified the counties in Colorado by their RUCC, and found that demolitions took place in urban counties at more than 10 times the rate of demolitions in rural counties; out of 28,403 possible demolitions, 26,011 occurred in rather urban counties (i.e., RUCC designations 1–5). We grouped RUCC 1–5 as urban counties and RUCC 6–9 as rural for all analysis using RUC codes. Comparing the total amount of demolitions occurring between 2008 and 2017 to the total number of built structures in 2015, we estimate that approximately 1.1% of Colorado’s building stock was demolished during this time period (thus an average annual rate of 0.11%). At the county scale we found, for both rural and urban counties, that the maximum percentage of demolished building stock did not exceed 2.5% during the 10-year period. As mentioned before, this observation also provides an estimated upper bound of potential commission error in ZTRAX and the derived land use datasets: There may be cases where demolished buildings are not reconstructed, and the demolition is not updated in ZTRAX, leading to a false positive (i.e., commission error). Furthermore, we refer to Uhl *et al*. (2021) where commission errors of ZTRAX-based settlement layers were quantified, and high levels of precision were observed in contemporary, urban settings, dropping to around 0.7 in rural settings and early time periods.

**Fig. 5:**
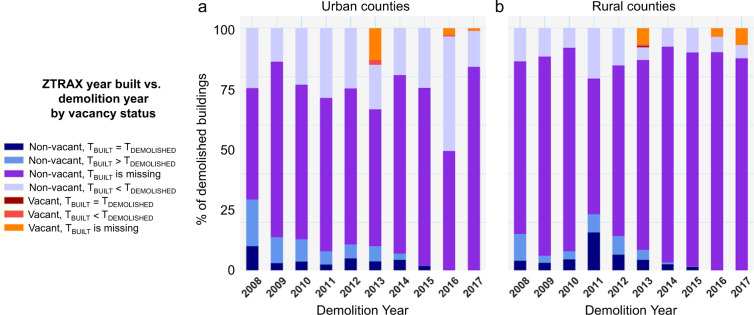
Cross-comparison of ZTRAX records and building demolition records in Colorado. The bar charts show the proportions of demolished buildings in different categories established by comparing demolition year and the year built on record in ZTRAX, separately for ZTRAX records reported as vacant and non-vacant. Urban counties have RUCC 1–5, rural counties have RUCC 6–9.

Moreover, we matched the demolition records to the ZTRAX records based on the address information given in both datasets and assessed the relationships between the demolition year and the year built on record in ZTRAX, separately for urban and rural counties (Fig. 5). The scraped demolition data contained a total of 33,645 addresses of which we were able to match 28,403 records to the ZTRAX data, leaving 5,242 records unmatched. These unmatched records may represent structures that have disappeared completely and are not contained in the ZTRAX data, or they are an artefact of our matching process, which was address-based and thus may be prone to misspelling errors in the addresses. We noted multiple instances in the scraped demolition data that were incorrectly spelled or had inconsistent formatting. From the analysis of this data we observed the following: (1) Most buildings that were demolished do not have a valid year-built attribute, and this proportion is higher in rural than in urban counties. This indicates that missing year-built attributes may be a result of recent teardowns, and possibly reconstruction, and a reporting latency that appears to be higher in rural counties. (2) Only a small percentage of demolished buildings (around 10% in 2008) have a year built > = year demolished. These records represent rebuilding activity that likely caused a replacement of the prior year built and represent the survivorship bias in ZTRAX- derived building age information. (3) Around 20% in 2008, and 50% in 2016 of demolitions did not cause an update of the year built on record in ZTRAX (year built <year demolished). This could imply several things: (a) The buildings were demolished and not replaced (i.e., they “disappeared”), but data records were not updated. This would illustrate an important limitation of our data, i.e., the shrinkage of human settlements cannot be measured. (b) The buildings were demolished and replaced, but the year built was not updated. This scenario would reduce the survivorship bias with respect to building age (i.e., the “original” year built persists); and (c) The buildings were demolished and replaced, and in addition to that, the building function changed: This would be an example of historical land use change not captured in our data. Furthermore, only a very small portion of demolished buildings is labelled as “vacant” in ZTRAX, indicating that most demolitions are followed by immediate reconstruction, or these cases are underreported in ZTRAX, which again would be an example of the inability to capture the shrinkage of built-up land in ZTRAX.

In conclusion, while we acknowledge the uncertainty due to survivorship bias contained in our data and generated by our modelling approach, it is clear that even in the most conservative scenarios that use verifiable data, survivorship bias would have minimal impact on analytical outcomes. As described above, we assume that land use for a structure was designated at the time the structure was built as this would have been the time that construction records/permits were submitted to the county assessor. Thus, some uncertainty remains unaddressed if buildings were built in parcels that have been re-zoned, and thus their land use designation may have changed, at some point in time. However, different kinds of land use changes over time have different transition likelihoods. We illustrate this in Table 7 to provide a basis for identifying land use classes that may be prone to this type of thematic uncertainty if past land use changes have not been recorded in the database.

**Table 7:** Estimated likelihoods of land use transitions over time.

		New land use type					
		RES-INCOME	RES-OWNED	COM	IND	AG	REC
	RES-INCOME		possible	possible	unlikely	unlikely	unlikely
	RES-OWNED	possible		possible	unlikely	unlikely	unlikely
	COM	possible	possible		unlikely	unlikely	unlikely
Initial land use type	IND	possible	possible	possible		unlikely	possible
	AG	possible	possible	unlikely	unlikely		possible
	REC	unlikely	unlikely	unlikely	unlikely	unlikely	

### Qualitative comparison to overhead imagery

**Fig. 6:**
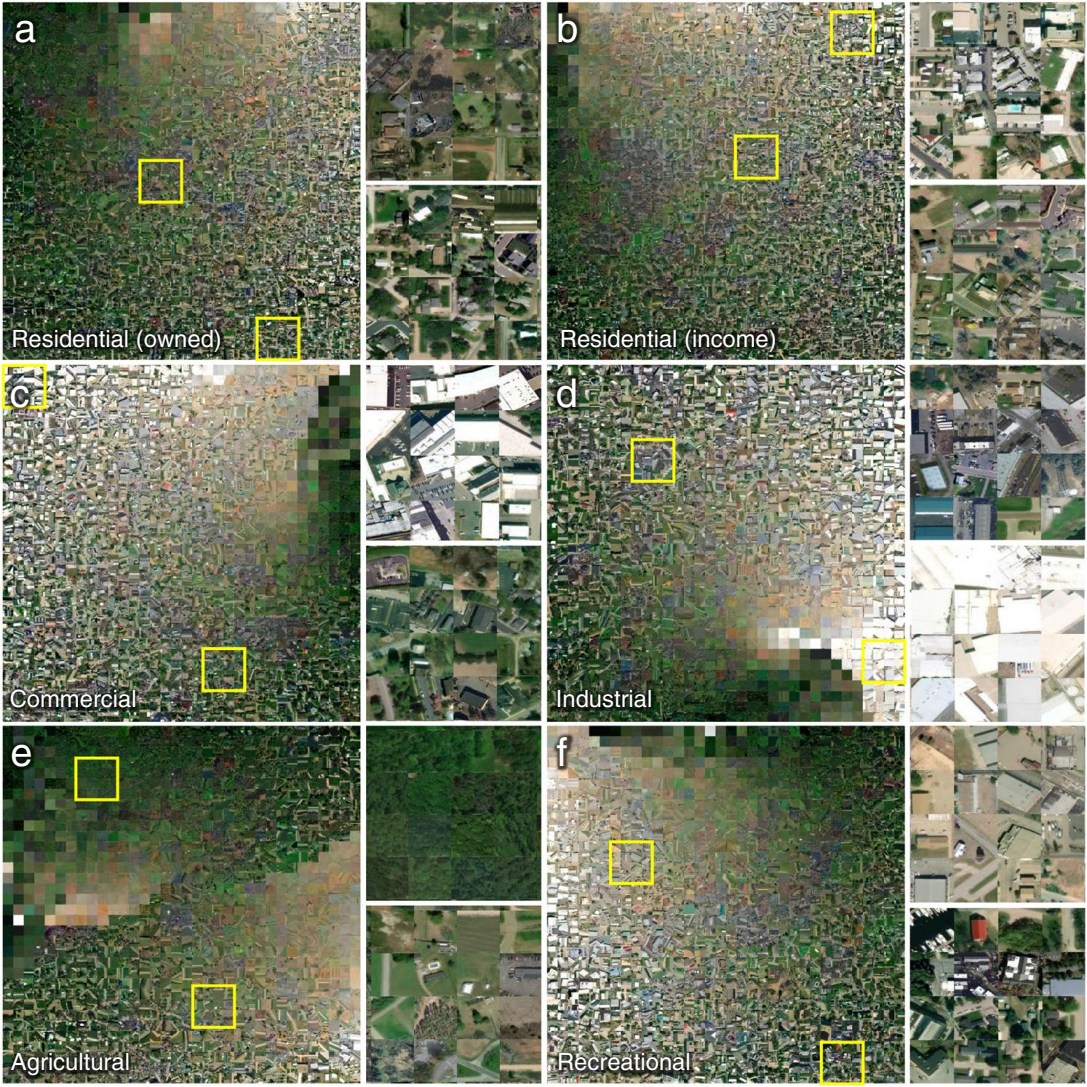
Visual assessment of Bing overhead imagery collected at the locations of a stratified random sample of ZTRAX records for the six land use classes used herein. (**a**) residential (owned), (**b**) residential (income), (**c**) commercial, (**d**) industrial, (**e**) agricultural, and (**f**) recreational. The images collected at each location per land use class are arranged based on their color similarity, using color moments and t-distributed stochastic neighbour transform (t-SNE). The small patches to the right of each mosaic show exemplary enlargements, providing further detail on the building characteristics at each ZTRAX location. The yellow rectangles show the locations of the enlargements (the upper enlargement corresponds to the upper of the two rectangles per land use class.

Lastly, we used Bing aerial imagery (https://www.arcgis.com/home/item.html?id=8651e4d585654f6b955564efe44d04e5) to qualitatively assess the relationship between broad-scale patterns of land surface in remotely sensed earth observation data and the different land use classes recorded in ZTRAX. To do so, we randomly selected one location per land use class, for each of the 3,019 covered counties, resulting in a total of 18,114 sample locations. We then obtained the RGB Bing imagery within a bounding box of 100 × 100 meters around each ZTRAX location, and generated a mosaic of these individual images, per land use class. These mosaics are based on a method proposed and employed in Uhl *et al*.^80^ which involves the calculation of color moments^81^ for each image, resulting in a 12-dimensional low-level descriptor summarizing the color content of each image. We then use t-distributed stochastic neighbour embedding (T-SNE)^82^ to map these 12-dimensional descriptors into a bi-dimensional space. T-SNE arranges data points in the bi-dimensional target space in a way that similar data points are located near each other. We then rectified the resulting 2-d point cloud and visualized each image at its corresponding location in t-SNE space. This method groups similar images together and allows for an integrated visual assessment of large amounts of images (Fig. 6). These visualizations characterize the broad-scale patterns of geographic contexts encountered at the ZTRAX locations per land use class. For example, they illustrate the quantity of vegetation-dominated settings, which are most frequent in the agricultural land use class. Small buildings are commonly found in the agricultural and residential land use classes. Large bright objects represent the (typically flat) roofs of large industrially, commercially, or recreationally used structures and seem to occur commonly at locations of these three land use classes. Note that images containing vegetation only are likely seen due to positional offsets of ZTRAX point locations from the actual building locations within rural (often larger) cadastral parcels^50^. However, we can assume that these offsets have a minor effect on the data accuracy due to the chosen spatial resolution of 250 × 250 m, as recent multi- scale accuracy assessments have suggested^24^. Thus, the visual inspection of the t-SNE plots in Fig. 6 reveals plausible matches for most sample locations. This technique could be used to systematically refine larger-scale samples for building level verification to conduct quantitative accuracy assessments, as far as building function can be inferred from overhead imagery.

In this analytical effort, we used OpenStreetMap, demolition data records, remote-sensing derived land cover classifications and overhead imagery as comparative data sources, being aware that none of these external data sources represent optimal ground truth to evaluate the quality of the created land use layers. While these comparisons do not quantify the uncertainty in historical land use data, they highlight important data quality aspects and properties that help to better understand the completeness and inherent bias in the data product.

## Usage Notes

In previous sections we have described our efforts to quantify certain biases that are present in the land use data. However, there are several other limitations that the user should consider when employing the gridded land use datasets. First, ZTRAX relies heavily on county records to populate the land use attributes, and county reporting practices differ from place to place, which may not account for all buildings that exist. Similarly, the implemented land use classification procedure may differ from county to county, which introduces some uncertainty related to the building type. We attempted to mitigate this uncertainty by grouping the 300 + land use types into broad thematic classes e.g., commercial or residential. A significant limitation of this dataset comes from the collection methods used to build the ZTRAX database; public buildings such as universities and low-income housing are generally not represented in the data presented herein. The gridded land use data will thus typically characterize privately owned structures. We recommend users integrate open-source data to capture the presence of public buildings within a given area to attenuate the error introduced by the exclusion of these buildings. Finally, we emphasize that this data was restricted to the land use of physical structures and the thematic classes that have been identified in the literature as significant to urban development. Thus, the data does not account for land uses that are not associated with a structure e.g., cropland or grazing land, and the data excludes other potentially important land use classifications such as tax exempt or governmental structures. As there has historically been a dearth of data that can directly describe structural land use in developed areas, the design of this data product intentionally gives preference to the land use classes identified as drivers of urban development at the expense of other potentially important land use types. Users should be aware of this inherent bias, and we encourage them to utilize the uncertainty layers to estimate the number of excluded structures within the considered study area.

## Data Availability

The ZTRAX dataset was stored in relational databases using Safe Software Feature Manipulation Engine (FME) (https://www.safe.com/). Code for this pipeline is available at https://github.com/johannesuhl/ztrax2sqlite2csv.
