# Author Correction: Gridded land use data for the conterminous United States 1940–2015

**DOI:** 10.1038/s41597-022-01642-6

**Published:** 2022-08-22

**Authors:** Caitlín Mc Shane, Johannes H. Uhl, Stefan Leyk

**Affiliations:** 1 Department of Geography, University of Colorado Boulder, 260 UCB, Boulder, CO 80309 USA; 2 Cooperative Institute for Research in Environmental Sciences, University of Colorado Boulder, Boulder, CO 80309 USA; 3 Institute of Behavioral Science, University of Colorado Boulder, Boulder, CO 80309 USA

**Keywords:** Environmental social sciences, Research data, Geography

Correction to: *Scientific Data* 10.1038/s41597-022-01591-0, published online 13 August 2022

The order of the tables on the in original version was incorrect, with tables published in the wrong positions and associated with the wrong captions. This has now been corrected as follows

The original Table 1 has been moved to the Table 4 caption

The original Table 2 has been moved to the Table 5 caption

The original Table 3 has been moved to the Table 6 caption

The original Table 4 has been moved to the Table 7 caption

The original Table 5 has been moved to the Table 1 caption

The original Table 6 has been moved to the Table 2 caption

The original Table 7 has been moved to the Table 3 caption

The Table 3 caption was also truncated, and has now been replaced with the full version “Cross-tabulation of land use (lu) and year built (by) completeness; “n” indicates missingness, e.g., nby = “no built year”.

These errors have been corrected in the HTML and PDF versions of the paper.

